# Analysis of air pollution over Hanoi, Vietnam using multi-satellite and MERRA reanalysis datasets

**DOI:** 10.1371/journal.pone.0196629

**Published:** 2018-05-08

**Authors:** Kristofer Lasko, Krishna Prasad Vadrevu, Thanh Thi Nhat Nguyen

**Affiliations:** 1 Department of Geographical Sciences, University of Maryland, College Park, Maryland, United States of America; 2 Earth Science Office, NASA Marshall Space Flight Center, Huntsville, Alabama, United States of America; 3 University of Engineering and Technology, Vietnam National University, Hanoi, Vietnam; Montana State University Bozeman, UNITED STATES

## Abstract

Air pollution is one of the major environmental concerns in Vietnam. In this study, we assess the current status of air pollution over Hanoi, Vietnam using multiple different satellite datasets and weather information, and assess the potential to capture rice residue burning emissions with satellite data in a cloud-covered region. We used a timeseries of Ozone Monitoring Instrument (OMI) Ultraviolet Aerosol Index (UVAI) satellite data to characterize absorbing aerosols related to biomass burning. We also tested a timeseries of 3-hourly MERRA-2 reanalysis Black Carbon (BC) concentration data for 5 years from 2012–2016 and explored pollution trends over time. We then used MODIS active fires, and synoptic wind patterns to attribute variability in Hanoi pollution to different sources. Because Hanoi is within the Red River Delta where rice residue burning is prominent, we explored trends to see if the residue burning signal is evident in the UVAI or BC data. Further, as the region experiences monsoon-influenced rainfall patterns, we adjusted the BC data based on daily rainfall amounts. Results indicated forest biomass burning from Northwest Vietnam and Laos impacts Hanoi air quality during the peak UVAI months of March and April. Whereas, during local rice residue burning months of June and October, no increase in UVAI is observed, with slight BC increase in October only. During the peak BC months of December and January, wind patterns indicated pollutant transport from southern China megacity areas. Results also indicated severe pollution episodes during December 2013 and January 2014. We observed significantly higher BC concentrations during nighttime than daytime with peaks generally between 2130 and 0030 local time. Our results highlight the need for better air pollution monitoring systems to capture episodic pollution events and their surface-level impacts, such as rice residue burning in cloud-prone regions in general and Hanoi, Vietnam in particular.

## Introduction

Biomass burning, industries, transportation, economic growth, and power production in Southeast Asia have been increasing in recent times, resulting in air pollution and air quality degradation issues throughout Southeast Asia [1–3]. Emissions from agricultural rice residue burning, forest biomass burning as well as industrial sources, have all been linked to long and medium range transport of air pollution in different regions of the world. For example, agricultural residue burning in the Indo-Ganges region resulting in pollutant deposition in the Himalayas with positive radiative forcing [4–5], arctic ice loss attributed to surface albedo change from pollutant deposition from agricultural fires in Russia [6], air quality degradation in Japan attributed to fires in Russia [7], cold season transport of industrial pollutants from China to Korea [8], degraded air quality in Singapore and Malaysia due to peat and palm plantation fires in Indonesia [9], as well as biomass burning pollution impacts in Thailand due to local and regional fires from Myanmar, Laos, and Cambodia [5, 10–14].

Black carbon (BC) is a key short-lived pollutant species paramount for air pollution abatement, and air quality, and it can also influence regional radiative forcing, precipitation, and monsoon patterns [4,15]. With the previous examples demonstrating the complex interplay and interaction of air pollution emissions throughout the world, BC is known to contribute to human health problems such as asthma, even with short-term exposure from episodic events such as agricultural residue burning [16]. BC surface concentration levels are especially influenced by winds, precipitation, planetary boundary layer, as well as atmospheric mixing heights due to sensitivity to wet and dry deposition [17–20]. Studies have also demonstrated that the effects from air pollution events can persist on a scale of days to months with impacts on atmospheric chemistry, weather, biogeochemical cycles, and linkage to disease and premature death [21–26]. However, monitoring pollutant impacts can be limited by uncertainty and discrepancies between air pollution datasets [27–28].

Of the different countries in Asia, Vietnam emits approximately 83Gg of BC annually, and it as the fifth highest emitter after some of the most populous countries including China, India, Indonesia, and Pakistan [3]. Much of the BC emissions from Vietnam are concentrated in the capital region of Hanoi, home to over 10 million people [29] with areas of forest biomass burning impacting air pollution in the Northwest, and rice straw burning within the Red River Delta surrounding Hanoi [30]. It’s uncertain how much of the air pollution in Hanoi is due to different sources from the surrounding region. This study explores how and if pollution contribution from other sources impact Hanoi’s pollution levels, such as local rice residue burning within the Red River Delta during June and October [31], as well as urban pollution from megacities in Southern China, and absorbing aerosols from forest fires in Laos and Vietnam [30].

In addition to BC, the Ultra-Violet Aerosol Index (UVAI) can monitor biomass burning events due to their release of absorbing aerosols associated with positive UVAI values [32–33]. However, use of this UVAI optical satellite data alone, can be difficult to accurately quantify pollution levels in cloud-covered regions such as Hanoi, Vietnam, as the satellite data can be obscured by cloud cover, although less so than other datasets due to use of UV-spectrum. It may also be difficult to quantify short, but intense biomass burning events such as from rice straw burning in June or October, as emissions may be focused across just several weeks with daily variability and low smoke plume heights. However, reanalysis data which assimilates datasets from a variety of platforms, provides mostly consistent spatiotemporal coverage through time, but with its own limitations.

In this study, we use a timeseries of reanalysis BC concentration data, as well as satellite-derived UVAI, MODIS active fires, and synoptic meteorology patterns to explore trends in air pollution across Hanoi, Vietnam, and the surrounding region to investigate potential sources of air pollution, as well as to determine if rice residue biomass burning events, which emit a large amount of absorbing aerosols, are captured in the timeseries of UVAI or BC data. We also assessed robust 3-hourly datasets of BC and rainfall-adjusted BC analysis based on the meteorology data. In this study, we addressed the following questions: 1) How does BC concentration in Hanoi vary across different time scales (i.e., hourly, daily and monthly)? 2) What are the major BC influencing factors? 3) How do the BC trends vary after adjusting for atmospheric conditions? 4) How well can rice residue burning emissions from the surrounding areas be linked with air pollution levels in Hanoi? (June and October).

### Study area

Hanoi, the cultural center and capital city of Vietnam, is located within the most populous urban area of the country, the Hanoi Capital Region, a substantial portion of the Red River Delta. The landscape includes a wide array of land cover types with the majority allocated to rice, impervious surfaces, and other croplands [34–35]. Hanoi is unique in that it is not only very populous with over 10 million people [29], but it exhibits a mosaic landscape covered with small-holder paddy rice, other farms, and plantations all intermixed amongst a growing peri-urban area resulting from ongoing conversion of agricultural lands [36]. The city has a humid, subtropical monsoonal climate influenced by the northeast monsoon during winter and the southeast monsoon during summer. Hanoi has the highest precipitation during summer (July-Aug) and lowest during winter (Dec-Jan). During many of the drier months, this most polluted city in Vietnam experiences routinely degraded air quality especially from fine particulate matter attributed to a variety of sources such as heavy vehicular traffic in Hanoi, rice residue burning during June and October, as well as regional transport from external sources [37–41].

## Data and methods

### Merra-2 reanalysis data

The Modern-Era Retrospective analysis for Research and Applications, Version 2 (MERRA-2) data is an improved, advanced data assimilation system combining hyperspectral radiance and microwave data, GPS-Radio occultation data, ozone profile observations, and several other datasets [42]. It is the first long-term global reanalysis dataset to include satellite-based aerosols and their interactions, with the aerosol emissions obtained from QFED, GFED, and RETROv2, but largely based on QFED for the temporal range of our study and BC concentration [42–46]. AeroCom Phase II dataset is used for anthropogenic emissions and is loosely based on year 1979–2006 observations. The aerosol species concentration is based on the Goddard Chemistry, Aerosol, Radiation, and Transport model (GOCART), and includes observations from NASA MODIS, MISR, and AERONET [47]. The dataset is available at approximately 50km spatial resolution similar to MERRA with a temporal resolution of 1-hour available from 1980-present. The data is freely available from NASA Goddard Earth Sciences (GES) Data and Information Services Center (DISC) in netCDF format. In this study, we obtained the 3-hourly data for 5 years from 2012–2016, and also processed it into corresponding day (9am– 12am), night (12am-9am), daily, monthly, and 3-hourly datasets useful for later comparison. Specifically, we obtained the Black Carbon Surface Mass Concentration (BCSMASS) subset and we processed it into ug/m^3^. In addition, wind vector data (u and v) were used to derive wind speed and direction using the following equations:

$$ws=\sqrt{u^{2}+v^{2}}$$

Where ws is wind speed in m/s and u and v are the wind vector components in the x and y direction respectively.

wd=atan2(v,u)

Where wd is the cardinal wind direction in degrees.

### Rainfall data

We obtained 5 years (2012–2016) of daily precipitation from Climate Hazards Group InfraRed Precipitation with Station (CHIRPS), version 2.0 data which combines coarse resolution satellite imagery with in-situ rain station data resulting in gridded rainfall data at approximately 0.05 degree spatial resolution [48]. We obtained netCDF rainfall data in mm/day and upscaled it to align and match the spatial resolution of MERRA-2 data. The CHIRPS data is freely available from 1981-present.

### Active fires data

For characterizing fire activity we used the MODIS collection 6 daily active fire hotspot data (MCD14ML) for 2012–2016 (also freely available for years 2000-present). The data comprises of fire locations derived from a contextual algorithm based on the thermal response of fire in the middle-infrared spectrum [49–50]. Fire hotspots are detected based on the MODIS instrument onboard the Aqua and Terra satellites with sun-synchronous, polar orbit passing over at approximately 1030am/pm and 130AM/pm local time. The MODIS Advanced Processing System processes the data using the enhanced contextual fire detection algorithm [49].

### Ultraviolet Aerosol Index (UVAI) data

The daily UVAI data available was obtained for 2012–2016 (freely available for years 2004-present). Positive UVAI values indicate the presence of absorbing aerosols such as dust, and also black carbon often associated with biomass burning activities. Whereas, negative values indicate non-absorbing aerosols. The OMEAERUV dataset based on the Ozone Monitoring Instrument (OMI) imagery in 354nm– 388nm spectral range produces UVAI measurements based on the columnar data and are provided at a spatial resolution of approximately 0.25 degrees [51–52]. Unlike MODIS with a two-satellite constellation, OMI onboard the Aura satellite, operates with one daily overpass instead of twice daily. Because UVAI values range from negative to positive with low values indicative of non-absorbing aerosols or cloud cover, we removed all values less than 0.1, in order to better represent absorbing aerosol pollution values related to biomass burning.

### Cloud cover data

Cloud cover data was freely obtained from NASA’s Clouds and the Earth’s Radiant Energy System (CERES) website. The SSF1deg dataset which contains MODIS cloud area fraction data was obtained for 12 years (2003–2014) at a daily temporal resolution and 1 degree spatial resolution, and freely available from 2000-present [53–54]. This dataset was used to provide ancillary information for the analysis and general cloud cover trends over Hanoi and the rest of the Continental Southeast Asia region.

## Methods

Hanoi, Vietnam has experienced routinely degraded air quality over the past several decades due to urban expansion and development, as well as emissions from rice residue biomass burning especially during June and October [31]. While emissions from rice residue burning have been quantified, it’s unknown how much impact is measured through the satellite air pollution datasets. We integrated meteorological factors such as wind speed, direction, and precipitation combined with MODIS active fire data to explore BC trends and levels. We also assessed the potential of UVAI for monitoring absorbing aerosols from biomass burning, such as rice residue burning in Hanoi, which is a heavily cloud-covered region. Moreover, we compare average monthly MODIS cloud fractions over the region, as well as the average monthly number of clear sky observations of OMI UVAI per month.

We employed a timeseries of 3-hourly Merra-2 Reanalysis BC concentration data to explore pollution trends over both Hanoi City as well as the surrounding continental Southeast Asia. We explore the BC concentration and distribution of values throughout the time period using boxplots and timeseries plots, as well as exploring patterns of extreme values through time using the 90^th^ and 95^th^ percentiles of daily averaged data. We explore the same using 3-hourly data to further explore trends in diurnal variation of BC, and compare day and night values by averaging from 9am– 9PM (Day) and 9PM– 9AM (Night). We further investigate trends in BC concentrations during the rice residue burning months of June and October to determine if BC levels are elevated during these significant biomass burning events which emit a large amount of fine-particulate matter [31]. We also investigate pollutant transport and contribution to variability in BC levels from the surrounding region based on synoptic wind direction patterns and biomass burning based on MODIS active fire products averaged on a monthly scale throughout the surrounding continental Southeast Asia region.

To better understand the type of pollutants observed during different months, we compare UVAI values with BC values to infer if the source of pollution can be attributed to biomass burning or not as positive UVAI values can be representative of absorbing aerosols related to biomass burning [33].

Lastly, we adjust the BC data based on meteorological parameters such as rain, and compare the original BC with rain, which impacts BC levels significantly due to increased wet deposition. Previous work has adjusted, or detrended, different aerosol species based on meteorological variables and often found non-linear relationship between the pollutant and precipitation [55–57]. Rainfall-adjusted values have the most utility for monitoring changes in long-term pollutant trends as well as other long-term studies such as relating to climate, aerosols, and evapotranspiration [58–59]. A best-fit regression is conducted on the BC and rainfall data resulting in a non-linear power-function fit as,

$$y=a*x^{b}$$

Where ‘y’ is the daily BC concentration, ‘a’ is a constant and ‘x’ is daily rainfall. The equation can be linearized by taking the base 10 log of both sides of equation to obtain:

$${log}_{10}(y){=log}_{10}(a)+b*{log}_{10}(x)$$

Then for each day of BC data we minimize the effects of rainfall by first calculating the residual:

$$residual=y_{i}-ŷ$$

Where ŷ is the predicted BC and *y*_*i*_ is the observed BC for that day. We then subtracted the residual from our original BC data to get rainfall-adjusted BC concentration useful to compare variation between the biomass burning months and non-biomass burning months and general longer-term trends.

## Results

### Cloud conditions over Hanoi

**Fig 1:**
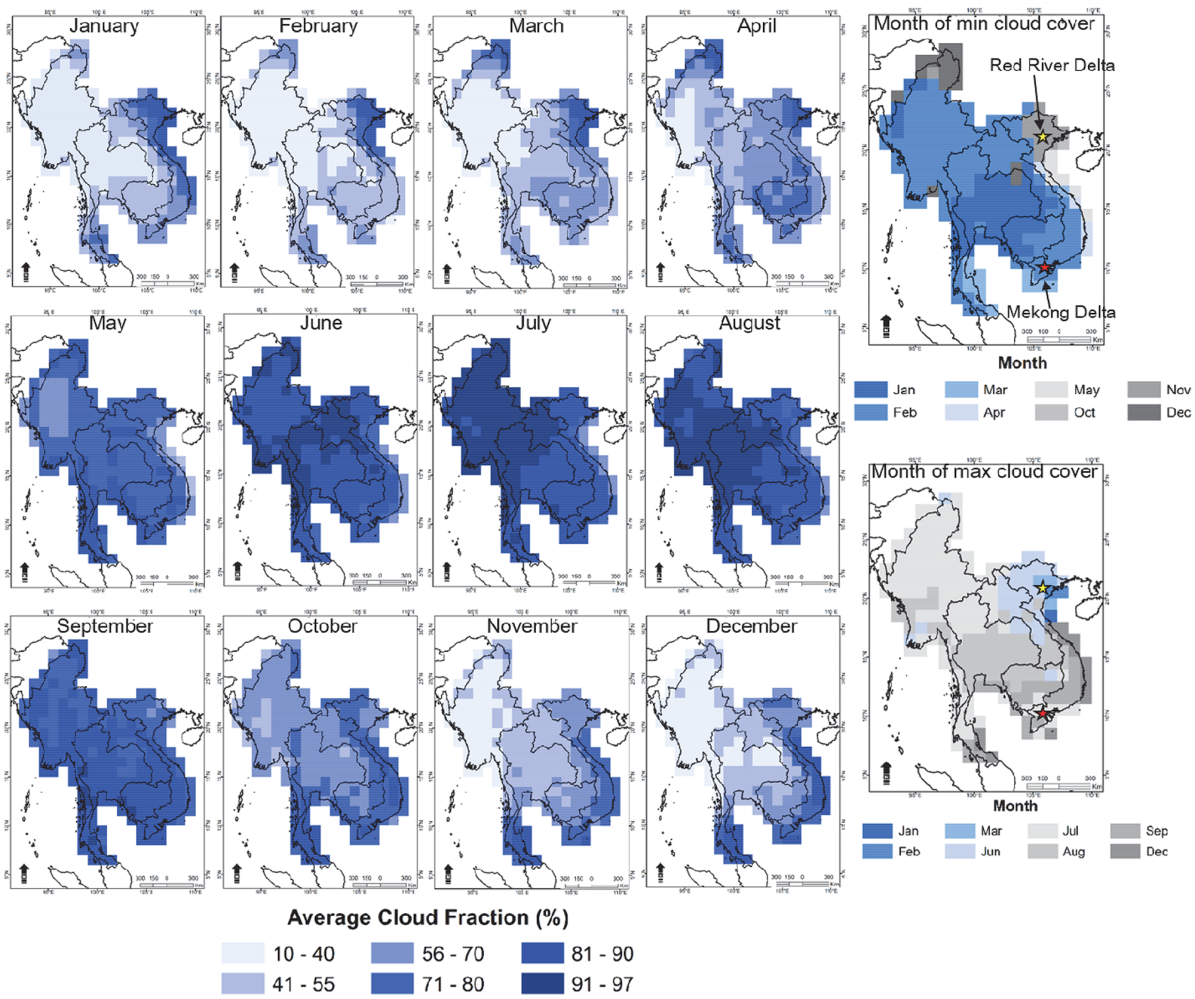
Monthly cloud cover averaged by month (2003–2014) per 1 degree grid cell.

Country-level analysis of cloud cover fraction in continental southeast Asia revealed Vietnam as having the highest monthly average cloud cover (72.4%) followed by Cambodia (69.7%), Laos (67.7%), Thailand (67.6%), and Myanmar (59.9%) (Fig 1). Moreover, of these different countries, Vietnam also has the lowest monthly active fire detections with 647 hinting at potential under detections due to cloud cover. Monitoring of active fires from rice residue burning in the Red River Delta is especially difficult. For example, the month of highest cloud cover is during the rainy season of July or August for most of Thailand, Myanmar, Cambodia, and Southern Vietnam. In contrast, Hanoi and the Red River Delta experience highest cloud coverage during the June residue burning time, whereas there are fewer clouds during November and December. In comparing the two rice growing regions of Vietnam, the Red River Delta averages only 25 active fires during the June rice burning time, whereas the Mekong River Delta averages 556 fires during the peak residue burning month of March. This discrepancy can be largely attributed to the higher cloud cover over the Red River Delta during burning months, compounded by relatively smaller field and fire sizes with average field size of about 5000m^2^ in the Mekong River Delta compared to about 800m^2^ in Hanoi and the Red River Delta [30–31, 60–61].

Consistently high cloud coverage over the Red River Delta and Hanoi, has important implications for monitoring air pollution and air quality in these regions. For example, current satellite-based datasets provide daily observations of global air pollution, but cannot provide meaningful local observation if obstructed by cloud.

### Wind and active fires

**Fig 2:**
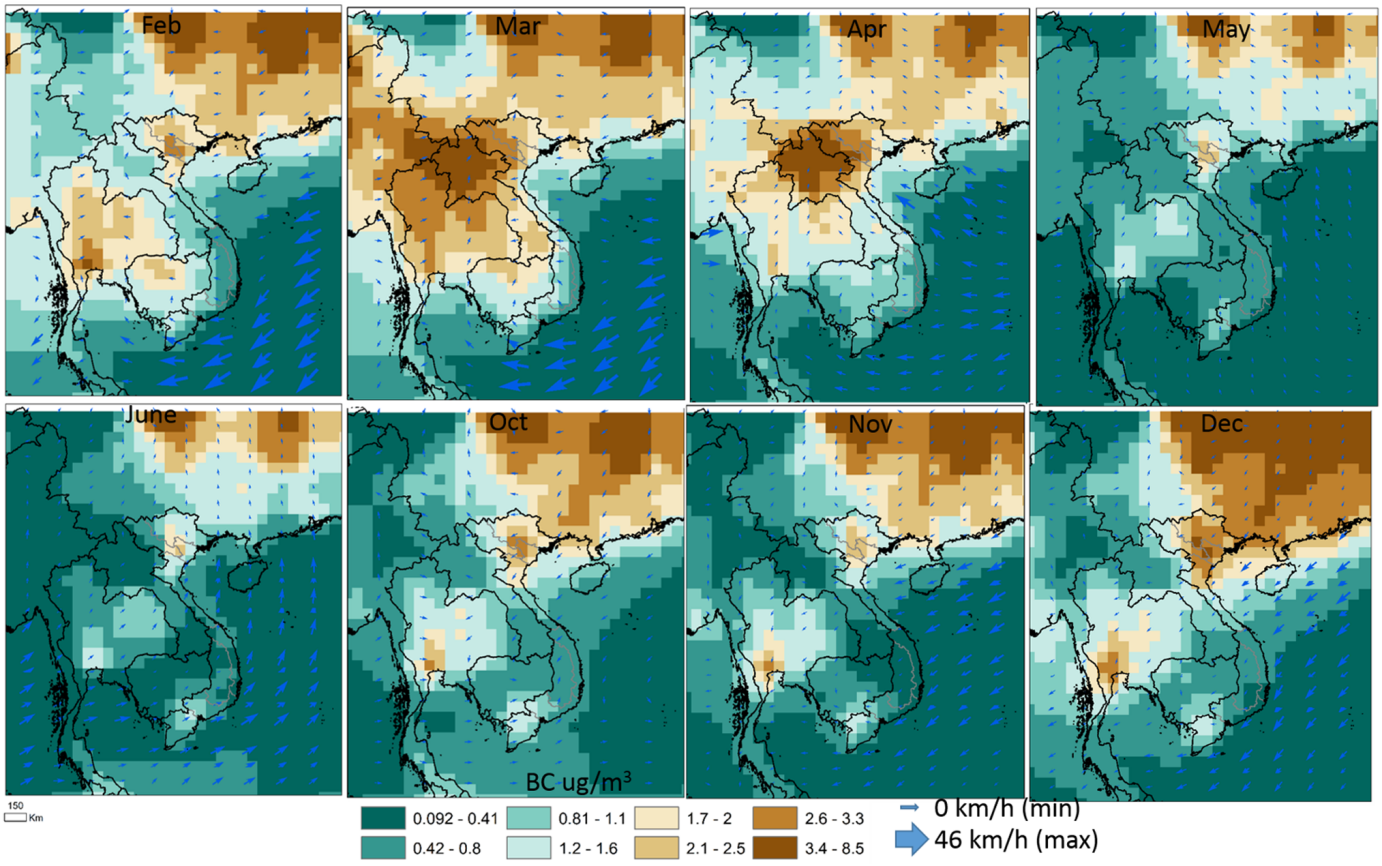
Monthly average BC concentration, wind direction and speed.

**Fig 3:**
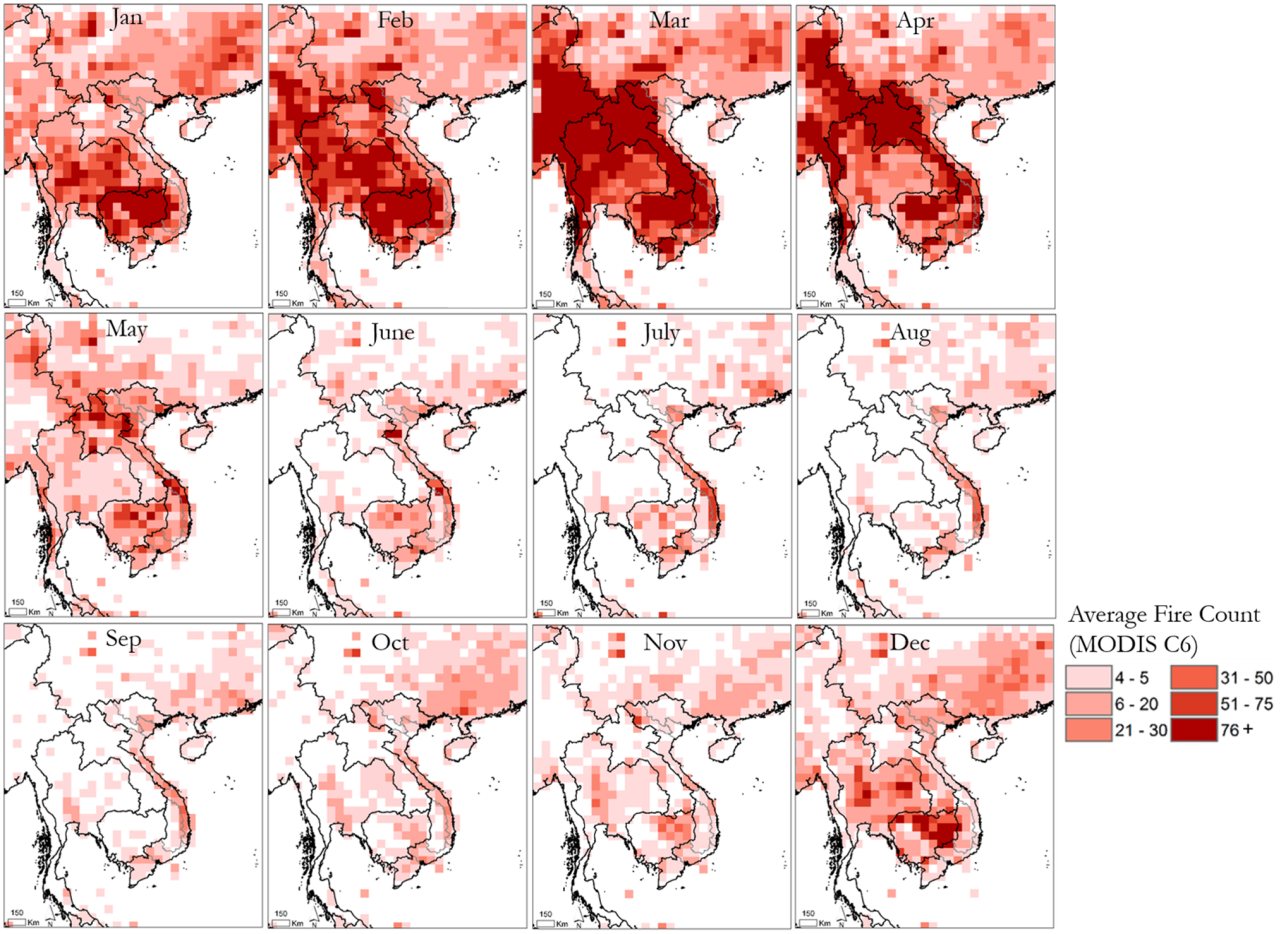
Average MODIS active fire trends in the surrounding region.

Transport of polluted air into Hanoi becomes apparent from monthly averaged wind direction and speed patterns shown in Fig 2 along with monthly average BC surface concentrations. General synoptic meteorology patterns indicate northerly winds flowing from South China into Northern Vietnam and Hanoi during October–March, whereas during Apr-Aug more south or southeasterly winds from Laos, Southern Vietnam, and the South China Sea. BC levels in the Southeast Asia region show a peak during March across Thailand and Myanmar attributed to agricultural and small-holder burning fires detected by MODIS (Fig 3) [33]. During March and April, high BC values over Laos and Northwest province of Vietnam are attributed especially to forest fires and slash and burn agriculture [30]. Relatively high BC levels throughout all months are consistently observed from Southern China largely originating from the mega-urban Chengdu and Chongqing cities with biomass burning also as a non-negligible emission source during these months [62–63].

Trends in the latest MODIS collection 6 active fire algorithm show peak burning in March and April as well as high levels of burning during Jan-Feb across much of Laos, Myanmar, Vietnam, Cambodia, and Northern Thailand due to forest and agricultural biomass burning (Fig 3). Relatively low active fires are detected across the region during June–November, a large portion of which is attributed to the rainy season (Table 1).

**Table 1:** Average monthly MODIS active fire counts within 1000km of Hanoi.

Month	Avg fire count	Location
Jan	22,795	Thailand, Cambodia
Feb	31,544	Thailand, Cambodia, China, N/E/W of RRD
Mar	68,618	Laos, Thailand, N/China, E China, NW Vietnam
Apr	43,402	Laos (MAX),NW Vietnam (MAX), N China, E China Low,
May	6,777	Laos (medium), N Vietnam (low/med), E China (Low), Thailand (low)
June	1,994	E China (low-Med), NCC Vietnam (med), E of RRD (low/med)
July	1,078	RRD fires, E China
Aug	1157	RRD, E china (low)
Sep	986	RRD, E China (low/low-med)
Oct	1,811	E China: Medium, NW Vietnam (low)
Nov	1,963	N China, E China (low-med), NW Vietnam (low)
Dec	7,598	E China (med-high), N China (med-h9gh), Thailand (med-low), NW Vietnam (med), E of RRD (med-high)

BC concentrations during October reach as much as 45% higher than the following month of November with the largest difference in 2015. On average, BC levels are 19% higher in October than November. These two months are relatively comparable due to synoptic wind patterns, rainfall, and active fires, as well as being adjacent in time.

### UVAI and number of clear observations

**Fig 4:**
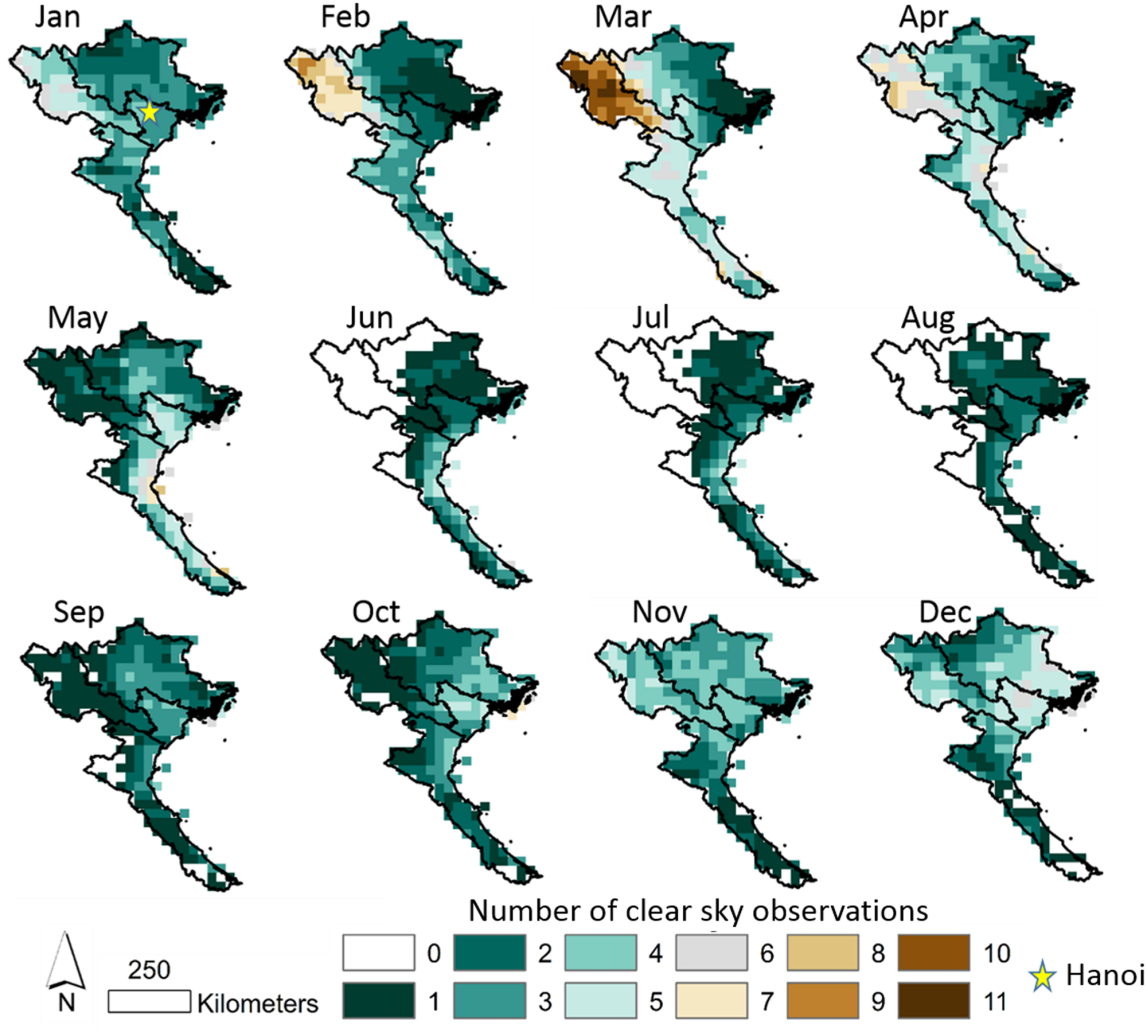
Average monthly number of non-cloudy UVAI observations over Northern Vietnam.

The UVAI is a satellite-derived pollution index sensitive to absorbing aerosols related to biomass burning. The average monthly total number of cloud-free observations for the UVAI (where UVAI > 0.1) are shown across Northern Vietnam in Fig 4. Over Hanoi, the highest number of observations occur during May and December which are generally drier months, still averaging only 5 or 6 clear observations. Whereas, very few observations are recorded especially during rainy months of June-August. The remaining months only experience between 1 and 5 clear observations on average (Fig 4). This lack of clear observations makes it very difficult to monitor the effects of air pollution with remote sensing. Moreover, because air pollution episodes often occur over rapid time-scales lasting for a few days [10,33], severe pollution events could be missed entirely, such as rice residue biomass burning events which occur in June and October.

**Fig 5:**
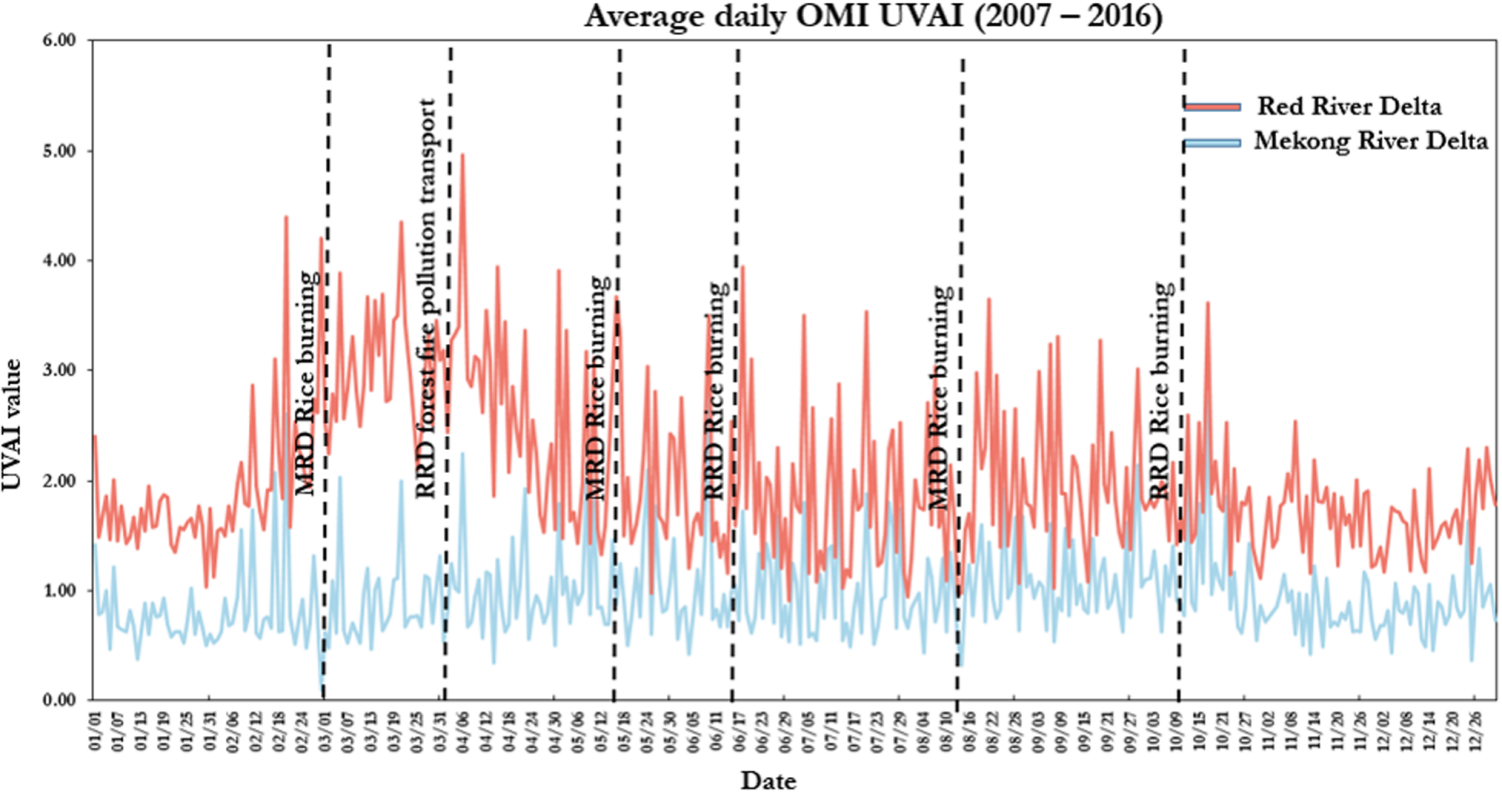
Average daily UVAI compared between Red River Delta and Mekong River Delta.

**Fig 6:**
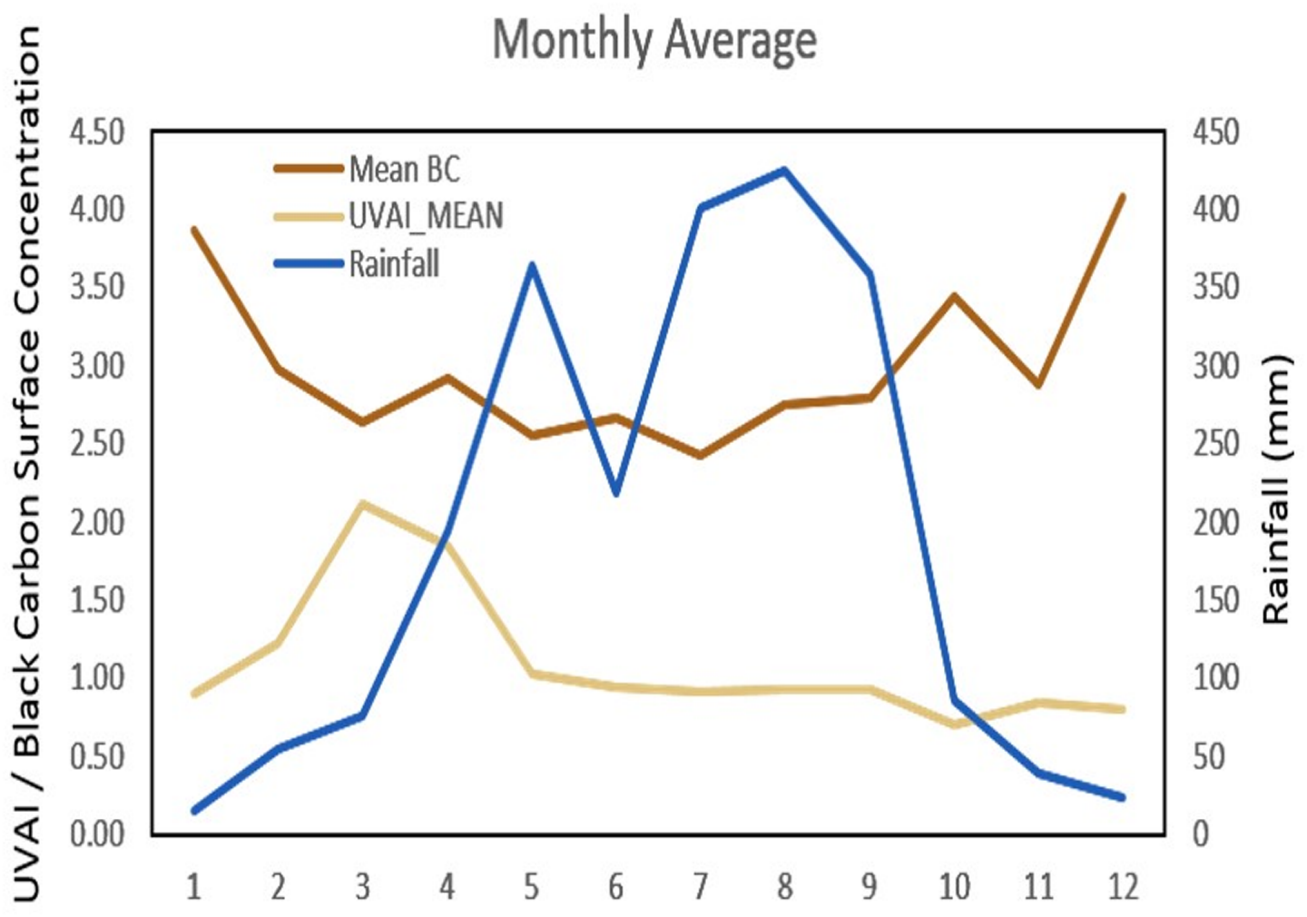
Monthly average BC, UVAI, and rainfall in over Hanoi, Vietnam.

To demonstrate an impact at least partially attributed to missing data, we show the daily average UVAI values averaged for the Red River Delta in Fig 5. A peak during March and April is observed where UVAI averages about 3.8 for both months. These are the peak months for air pollution over the region, and it can largely be attributed not only to local sources of pollution, but especially to transport of absorbing aerosols from forest fires in the Northwest of Vietnam and Laos. The latter will be demonstrated in a subsequent section. Aside from the peak during March and April, average monthly values for UVAI only range from 1.2 in December to 1.9 in August showing minimal monthly variation. At a daily-scale, very high values are occasionally observed during the other months such as October when rice residue burning is present (Fig 5). However, even though a lot of absorbing aerosol pollutants are emitted, monthly average observations do not demonstrate an increase in UVAI during residue burning months of June or October–suggesting the emissions impact may be entirely missed by satellite data [31], as shown in Fig 6 with rainfall and BC.

### MERRA-2 BC patterns and weather adjusting

**Fig 7:**
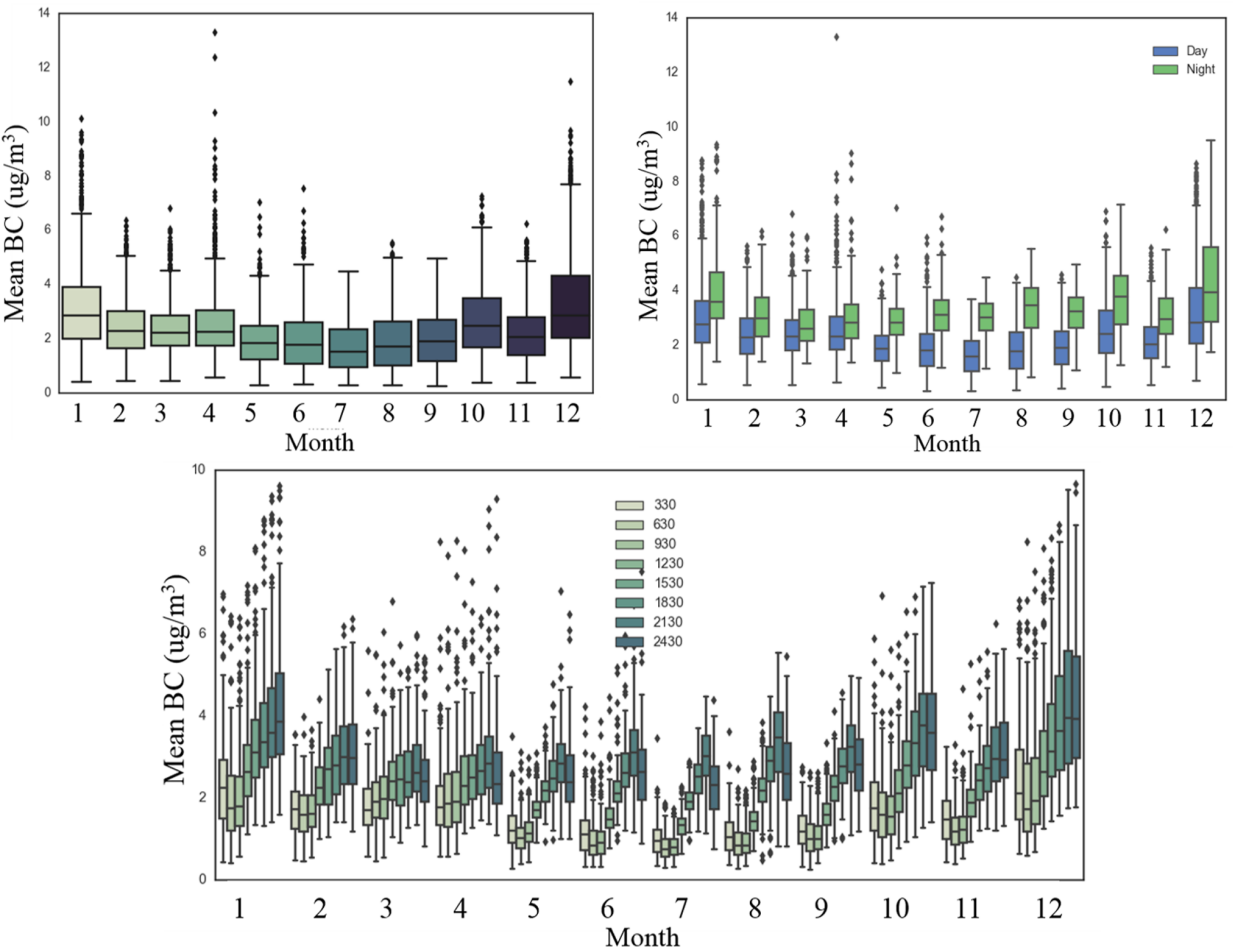
Distribution of 3-hourly BC surface concentration a) per month, b) day vs night; c) 3-hourly.

Because of the cloud cover issues over Hanoi, we use MERRA-2 reanalysis BC concentration data which uses a combination of satellite and ground-based datasets which provides data for every day regardless of clouds, however, observations are still effected by cloud cover. The MERRA-2 BC concentration data at 3-hourly intervals showed more dynamic trends in pollution than those observed through UVAI alone. The 3-hourly data analysis for each month revealed December (4.08ug/m^3^), January (3.87ug/m^3^), and October (3.44ug/m^3^) with the highest median and average monthly values. However, it should be noted that April often experiences the most outlier values indicating a large amount of pollution episodes (Fig 7). We also compared the distribution of pollution across day and night (Fig 7). The results illustrate higher nighttime BC concentrations than daytime values. In addition, the most diurnal variation occurs during rainy season months of June–September. Minimal diurnal variation has been observed during March and April with a difference of about 0.3ug/m^3^ between daytime and nighttime median values for March. Low diurnal variation may be indicative of large-scale, persistent biomass burning fires such as from Laos or NW Vietnam where pollutants could be transported through day and night, as compared with urban pollutants such as from traffic, which are more cyclic exhibiting diurnal variation. Moreover, diurnal variation from rainfall would also have an effect due to wet deposition, however, the rainfall observations are only available once per day.

Analysis of trends using 3-hourly monthly datasets showed the lowest BC values over Hanoi around 3:30am or 6:30am attributed to less vehicular traffic and industrial pollutants (Fig 7). BC concentration during the evening and nighttime are at least 200% higher than morning concentrations during June, July, August, and September. During the rice residue burning month of June, BC values are not elevated and are relatively similar to neighboring months of May and July. Whereas, during the other rice residue burning month of October, BC concentrations are slightly elevated and about 0.5ug/m^3^ higher at each 3-hour period than September–attributed to relatively lower rainfall. BC concentrations are about 19% higher in rice residue burning month of October than in November. Over the 5-year time period on a daily level, the BC concentrations indicated very slight decrease with y = -0.003x+2.7142 partially attributed to higher rainfall events in 2015 and 2016.

**Fig 8:**
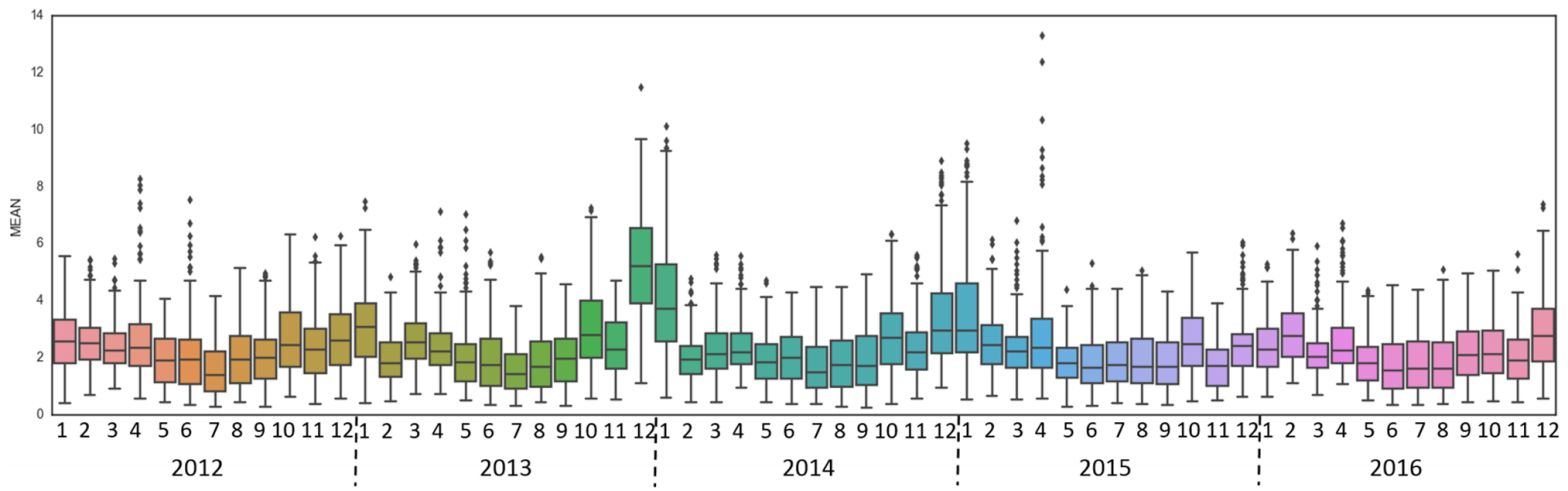
Monthly distribution of 3-hourly surface BC.

Timeseries distribution of the 3-hourly data for every month shows a cyclical pattern of BC levels aligned with the monsoon and associated rainfall. Abnormally high BC concentrations were observed during December 2013 and January 2014 with median values of 5.3ug/m^3^ and 3.7ug/m^3^ respectively (Fig 8). This severe pollution episode may be linked with pollution in South and East China with the pollutants transport from industrial and vehicular sources from mega-urban areas of Chongqing as shown in Fig 2 [62–63]. Since, UVAI had a peak only in March and April, we infer that biomass burning impacts over Hanoi are most significant during this time. However, we also know that absorbing aerosols from rice residue burning are emitted in June and October [31], but no increase in UVAI is observed during these months suggesting the satellite-based datasets miss this phenomena over Hanoi. More research with PM_2.5_ data may provide insights. In addition, because UVAI is not high during the peak BC months of January and December, we infer that these months may especially be impacted by non-absorbing aerosols from non-biomass burning including transport from other regions (i.e. southern China) and reduced rainfall effects, however the results are inconclusive due to limited UVAI observations in any given month.

**Fig 9:**
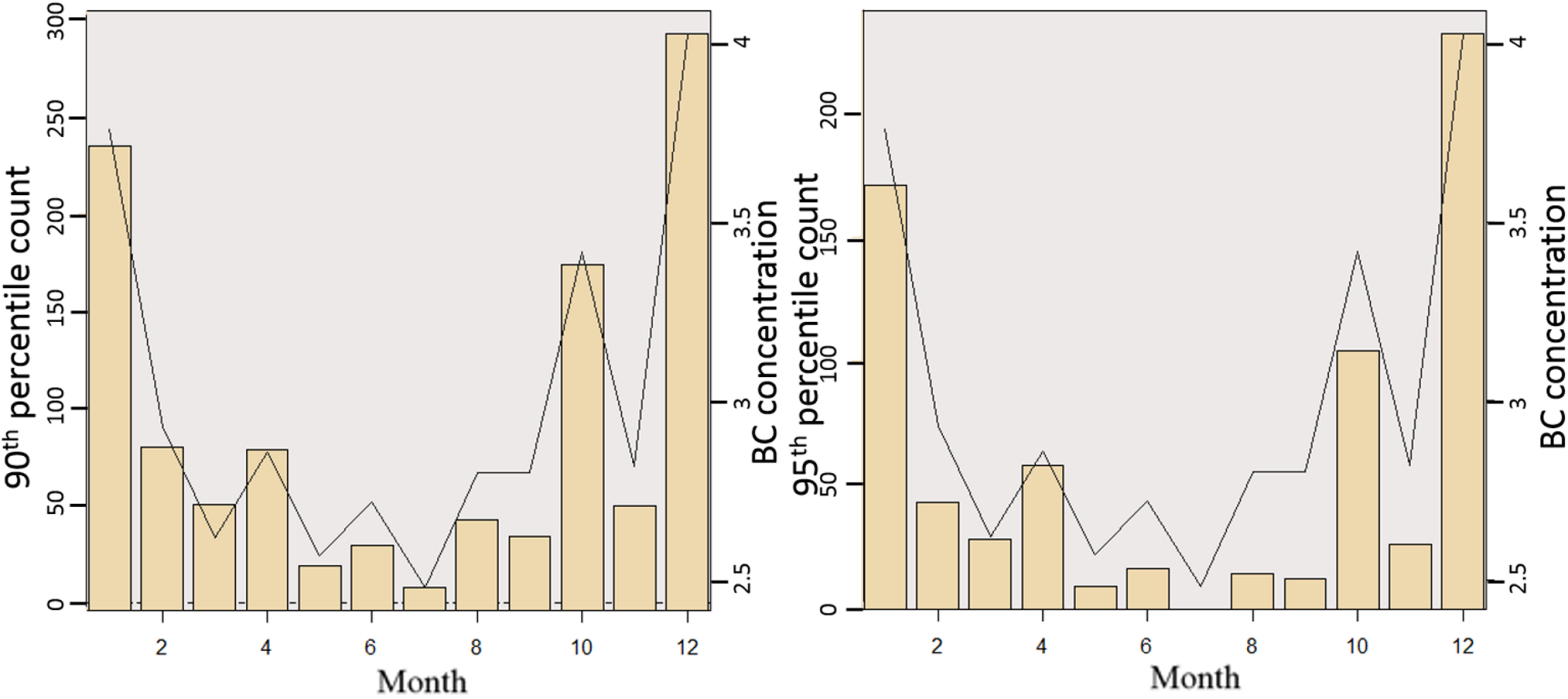
Number of 3-hourly exceedance of a) 90^th^ percentile; b) 95^th^ percentile. Black line represents monthly average BC.

While monthly averages and distribution of 3-hourly data provides some insight, they do not highlight temporal characteristics of extreme pollutant events. To better understand extreme pollution events in each month, we explored the exceedance of BC levels above 90^th^ and 95^th^ percentile (Fig 9). As expected, results showed most extreme values during December, January, and October followed by April, February, and March. In comparison to the rice residue burning months, June has less than 25 incidences exceeding the 90^th^ percentile, whereas October has about 170 suggesting that October may have more residue burning impacts than June.

The UVAI values peak during March and April, but are relatively stable in the remaining months. We infer that March and April have the highest air quality impacts due to absorbing aerosols emitted from biomass burning such as those transported from Laos and Northwest Vietnam as described in the subsequent section. Whereas, during the remaining months, low UVAI is observed. This includes low UVAI during the peak MERRA-2 BC levels in December and January. We attribute the MERRA-2 BC peak in December and January to non-absorbing aerosols from urban sources such as those transported from North of Hanoi and cities in southern China. Elevated UVAI levels are not observed during known rice residue burning months of June and October.

### Rainfall

Monthly rainfall patterns show a peak during July and August averaging 401 and 425mm respectively. Whereas October–March are relatively dry ranging from 84mm in October to 14mm in January (Fig 6). Over the five year period we observed a slight increasing trend in rainfall. For comparison purposes and to explore rice residue burning impact, we compare October (84mm rain) and November (39mm rain) due to similar rainfall, wind, active fires, and proximity. We note June (218mm rain) is more difficult to analyze air pollution concentration due to much different rainfall than adjacent months, therefore the pattern may be observed in de-trending. Moreover, the higher rainfall in June may contribute to relatively lower impact from rice residue burning than during October as seen in the BC data.

**Fig 10:**
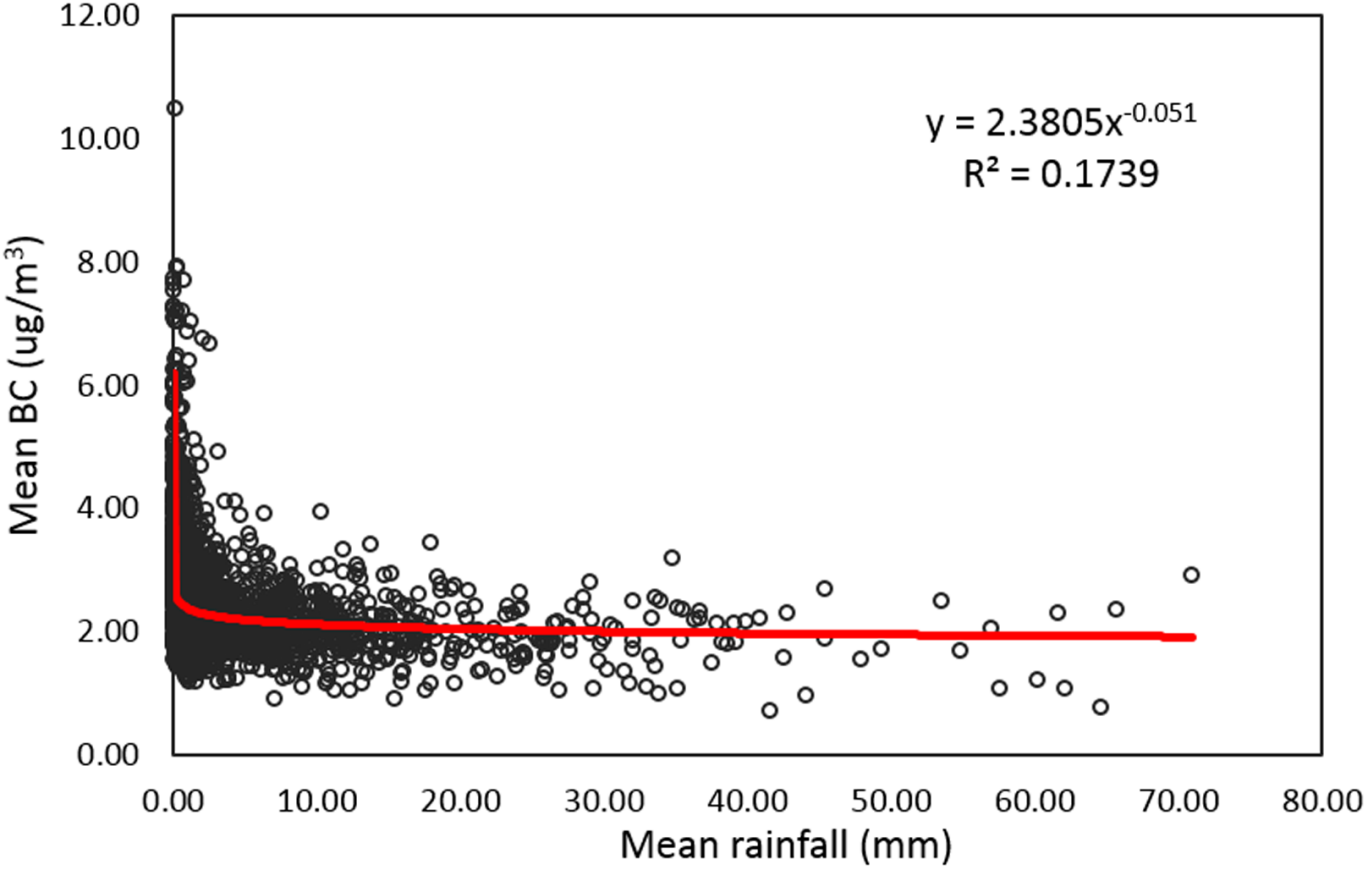
Relationship between daily rainfall and average BC over Hanoi, Vietnam.

**Fig 11:**
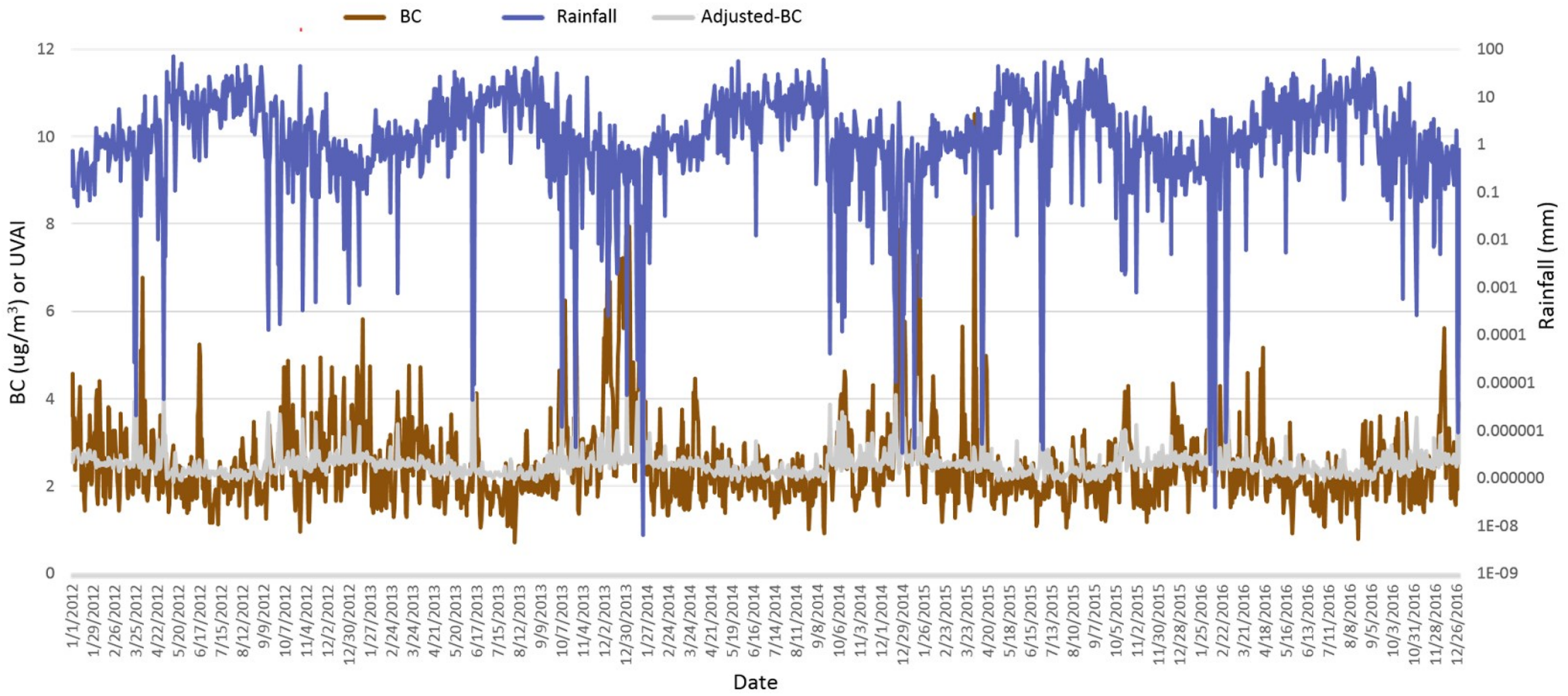
Timeseries of BC, rainfall, and weather-adjusted black carbon over Hanoi, Vietnam (2012–2016).

We adjusted the timeseries BC data based on the rainfall data over Hanoi to yield weather-adjusted BC values. Results show a negative non-linear power function trend where BC decreases as rainfall increases with about 17% of variation in BC attributed to rainfall (Fig 10). Results show the weather-adjusted values are much less variable through time and that BC values are much lower in many months such as June, July, and August due to rainfall events (Fig 11). However, the de-trending reduced overall monthly variation in the BC dataset with lowest concentration during August at 2.3ug/m^3^ and highest in January with 2.76ug/m^3^. The highest standard deviation of the daily BC data is during February, January, and October respectively ranging from 0.45 to 0.40ug/m^3^.

## Discussion

Previous studies have demonstrated that even limited duration exposure to polluted air such as PM_2.5_ or one of its major constituents of BC, may lead to serious health concerns and even premature death in at-risk populations such as the elderly [64]. One such example in Beijing estimated 5,100 people died prematurely due to air pollution exposure from 2001–2012 [65]. Therefore, it is of significant importance to address and properly quantify some of the air quality issues over Hanoi, such as from rice residue biomass burning events in June and October. However, it is also important to quantify air pollution for much of the Asian region due to long-range transport from different emission sources. These reasons highlight the need to mitigate air pollution emissions throughout the Asian region.

Our study results align well with the earlier studies conducted in Southeast Asia on air pollution and air quality trends. For example one recent study showed Laos, Thailand, Cambodia, and Myanmar emit large amount of BC emissions from non-agricultural fires [66]. Moreover, previous work has also found biomass burning emissions peak during March and April across continental Southeast Asia attributed to forest and agricultural burning [67]. A recent study in Thailand demonstrated peak biomass burning in the dry season of April not only due to fires in Northern Thailand, but also due to emissions from surrounding Myanmar, Laos, and India [13]. Previous studies have also demonstrated difficulty to model and monitor serious air pollution events in the Southeast Asian region [68], including those from rice residue biomass burning [31]. We found the BC values observed over the Hanoi area as relatively higher through time than other Southeast Asian countries such as Laos, Thailand, and Cambodia. Continued monitoring and collaborative efforts are critical to address the ongoing air pollution concerns in the region [69]. In the study region, capturing pollution trends coinciding with rice residue burning during June and October/early-November is difficult and confounded by smoke injection heights, highly variable rainfall and wet deposition, as well as a current lack of good quality surface concentration datasets.

Previous *in situ* studies in Hanoi have found the most polluted months to be during the December-February dry season, corroborating some of the findings of this study with peaks during January, December, and October [40]. Another study found that nocturnal radiation inversions during October–December and subsidence temperature inversions from January to March help to amplify air pollution and air quality concerns in Hanoi; as well as high pressure systems over Central China during winter leading to southerly air flow, additionally corroborating some of this study’s findings [37]. Moreover, research has also demonstrated polluted air parcel movement from southern and Eastern China during months of September–March, and pollutant transport from the Indochina peninsula during rainier months of June–August [38]. While the Hanoi area experiences monsoon-influenced conditions with heavy rains during the summer, studies have found that severe and strong fires can result after heavy rain events [70]. Another study demonstrated Hanoi ozone levels and NO_2_ levels as routinely exceeding WHO standards attributed to high levels of motorcycles and traffic [71]. However, a clear pattern from both UVAI and BC data relating to the large amount of rice residue burning practiced during June and October was not clear. Rice residue burning fires are typically small, low temperature fires difficult to monitor by satellite, especially due to varied burning practices [72]. Moreover, the UVAI data is a columnar measurement which does not capture surface concentration, while the BC data is similarly limited and often affected by interpolation issues. The MERRA-2 reanalysis data has some utility for monitoring air pollutant trends over time, but with caveats for rice residue and biomass burning emissions.

Our study demonstrated that MERRA-2 BC concentration is useful for capturing variation in air pollution concentration especially attributed to urban sources including transport from southern China especially during the dry season months of December and January. We found that Hanoi suffers elevated UVAI during March and April attributed to forest biomass burning transport from NW Vietnam and Laos, but no increase in the MERRA-2 BC is observed during this time. While we know from previous studies that rice residues are subjected to burning in June and October, emitting a large amount of PM2.5, both of these datasets did not detect elevated air pollution levels. It’s possible that this can be attributed to coarse spatial resolution datasets, lack of ground observations, as well as cloud cover obstructing satellite measurements. We found an average range of 2–5 clear UVAI observations in any given month making episodic biomass burning difficult to detect.

Although the MERRA-2 data has potential, it is limited by coarse resolution which may impede ability to monitor rice residue burning, confounded by emissions released steadily over the course of 4–6 weeks, diluting the signal. The MERRA-2 data, currently, is limited in effectiveness for rice residue burning emissions monitoring. The BC concentration dataset may not be sensitive to these biomass burning events considering BC includes a range of particle mass sizes and types, whereas rice residue burning events largely emit fine-particulate matter. Additionally, BC values may be relatively low in absolute terms as they are representative of a 0.5degree cell average which spreads across not only the Hanoi urban area, but also much of the rice areas and countryside resulting in slightly smoothed, averaged values. Moreover, there is a need for ground station datasets for improved air pollution monitoring, which would also be useful for comparison with MERRA-2 [73].

Future work should be done to simulate air pollutant concentrations resulting from rice residue burning to better understand the land-atmospheric interactions. Another future improvement can be through refined PM_2.5_ concentration data (not just PM_2.5_ dust as currently available) in MERRA-2 as it is more sensitive to rice residue burning due to its absorbing nature [74–75]. For example, PM_2.5_ concentration increases have been linked to rice residue burning events in China [76].

## Conclusion

Our study explored the current status of air pollution and pollution monitoring over Hanoi, Vietnam and the surrounding region, and specifically, to determine if the satellite data can capture the air pollution impact from rice residue biomass burning events documented to occur in the Red River Delta where Hanoi is located. Our results showed the Red River Delta and Hanoi experience the highest cloud coverage of the continental Southeast Asian region, and it was found to hinder optical satellite observations of air pollution, such as from OMI UVAI averaging only a few clear observations per month. Therefore, it is difficult to monitor air pollution in the study area and we did not find significant air pollution impacts over Hanoi attributable to the known rice residue burning events. Further improved methodologies or higher resolution satellite constellations, and ground-based datasets are needed for improved air pollution assessments. We also used MERRA-2 reanalysis data which found the highest BC levels during December, January, and October during the dry season with some pollution attributed to long range transport from the North during this time. We also found biomass burning emissions impact based on UVAI levels with transport from Laos and NW Vietnam during March and April based on synoptic wind patterns. Results from both UVAI and BC did not indicate elevated pollution levels from rice residue burning during June, however, slightly elevated levels in October were often observed. Findings suggest that improved datasets and observations are necessary for monitoring rice residue burning emissions.
