# Correction: Analysis of air pollution over Hanoi, Vietnam using multi-satellite and MERRA reanalysis datasets

**DOI:** 10.1371/journal.pone.0214628

**Published:** 2019-03-26

**Authors:** Kristofer Lasko, Krishna Prasad Vadrevu, Thanh Thi Nhat Nguyen

In the Funding statement, the grant number from Vietnam National Foundation for Science and Technology Development is incorrect. The correct grant number is: 105.08–2017.11.
